# Consensus on the definition, components, timeframe and grading of composite outcome of postoperative pulmonary complication—protocol for an international mixed-method consensus study (PrECiSIOn)

**DOI:** 10.1136/bmjopen-2025-103888

**Published:** 2025-08-19

**Authors:** Prashant Nasa, Timur Yurttas, Denise Battaglini, Stijn Blot, Ana Fernandez-Bustamante, Marcelo Gama de Abreu, David M van Meenen, Sheila Nainan Myatra, Ary Serpa Neto, Raymond Oppong, Frederique Paulus, Suresh Renukappa, Marcus J Schultz, Arthur S Slutsky, Sabrine N T Hemmes, for the PrECiSIOn–group

**Affiliations:** 1 Department of Intensive Care, Amsterdam UMC, location 'AMC', Amsterdam, The Netherlands; 2 Department of Critical Care Medicine and Anaesthesia, The Royal Wolverhampton NHS Trust, New Cross Hospital, Wolverhampton, UK; 3 Division of Anesthesiology, Rescue and Pain Medicine, HOCH Health Ostschweiz, St. Gallen, Switzerland; 4 Anesthesia and Intensive Care, IRCCS Ospedale Policlinico San Martino, Genoa, Italy; 5 Department of Surgical Sciences and Integrated Diagnostics, University of Genoa, Genoa, Italy; 6 Department of Internal Medicine and Pediatrics, Ghent University, Ghent, Belgium; 7 Department of Anesthesiology, University of Colorado System, Denver, Colorado, USA; 8 Departments of Intensive Care and Resuscitation and Outcomes ResearchDepartment of Cardiothoracic Anaesthesiology, Cleveland Clinic, Cleveland, Ohio, USA; 9 Pulmonary Engineering Group, Department of Anaesthesiology and Intensive Care Medicine, University Hospital Carl Gustav Carus, Technical University Dresden, Dresden, Germany; 10 Department of Anaesthesiology, Amsterdam University Medical Centres, Amsterdam, The Netherlands; 11 Department of Anaesthesiology, Critical Care and Pain, Tata Memorial Hospital, Homi Bhabha National Institute, Mumbai, Maharashtra, India; 12 Australian and New Zealand Intensive Care–Research Centre (ANZIC–RC), Monash University, Melbourne, Victoria, Australia; 13 Department of Intensive Care, Austin Hospital, Melbourne, Victoria, Australia; 14 Department of Critical Care Medicine, Hospital Israelita Albert Einstein, São Paulo, Brazil; 15 Health Economics Unit, Department of Applied Health Sciences, College of Medicine and Health, University of Birmingham, Birmingham, UK; 16 Faculty of Science and Engineering, University of Wolverhampton, Wolverhampton, UK; 17 Mahidol Oxford Tropical Medicine Research Unit, Mahidol University, Bangkok, Thailand; 18 Nuffield Department of Medicine, University of Oxford, Oxford, UK; 19 Department of Anaesthesia, General Intensive Care and Pain Management, Division of Cardiothoracic and Vascular Anaesthesia and Critical Care Medicine, Medical University of Vienna, Vienna, Austria; 20 Interdepartmental Division of Critical Care Medicine, University of Toronto, Toronto, Ontario, Canada; 21 Li Ka Shing Knowledge Institute, St. Michael’s Hospital, Toronto, Ontario, Canada; 22 Department of Anaesthesiology, The Netherlands Cancer Institute – Antoni van Leeuwenhoek Hospital, Amsterdam, The Netherlands

**Keywords:** ANAESTHETICS, Delphi Technique, Adult anaesthesia

## Abstract

**Introduction:**

Postoperative pulmonary complications (PPCs) represent a significant cause of postoperative morbidity and even mortality. However, there is a lack of consensus regarding this composite endpoint, the definition of the individual components, classification and optimal outcome measures. This study aims to refine the PPCs composite framework by evaluating its construct validity, assessing the necessity and risks of a composite measure and exploring the feasibility of differentiating severity categories.

**Methods:**

A Delphi consensus process will be conducted, engaging an international multidisciplinary group of 30–40 panellists, including clinicians, researchers, patients, public representatives and health economists. Through iterative rounds, the study will seek agreement on the individual components of the PPCs composite. Additionally, consensus will establish a framework for a composite outcome measure based on a standardised severity classification, appropriate timeframes and weighted grading of PPCs.

**Analysis:**

Consensus, defined by ≥75% concurrence in multiple choice questions or on Likert–scale statements, will be evaluated from round 2 onwards. Delphi rounds will be continued until all statements have reached stability of responses evaluated by χ^2^ tests or the Kruskal-Wallis test.

**Ethics and dissemination:**

The study will be conducted in strict compliance with the principles of the Declaration of Helsinki and will adhere to ACCORD guidance for reporting. Ethics approval has been obtained for this study from the University of Wolverhampton, UK (SOABE/202425/staff/3). Informed consent will be obtained from all panellists before the commencement of the Delphi process. The results of the study will be published in a peer–reviewed journal with the authorship assigned in accordance with ICMJE requirements.

**Trial registration number:**

NCT06916598 (clinicaltrials.gov).

**STRENGTHS AND LIMITATIONS OF THIS STUDY**

- This Delphi study will engage a diverse and international panel of healthcare workers from Anaesthesiology, Surgery or Intensive Care Medicine involved in the management of patients with postoperative complications, as well as patients and patient care representatives, and health economists.
- The Delphi process will ensure the anonymity of the panellists and their voting process on the defining PPCs, to avoid any potential peer bias or group conformity.
- Variations in patients’ and patient care representatives’ preferences and differences in local or regional guidelines may influence panellists’ interpretation of statements and their opinions.

## Introduction

 Development of one or more postoperative pulmonary complications (PPCs) is linked to increased hospital length of stay and higher mortality rates,^1 2^ as well as substantial clinical and economic implications.^3 4^ Over the past decade, PPCs have increasingly been used in studies focussing on preventive perioperative measures, such as lung–protective intraoperative ventilation and postoperative respiratory support.^5^ The incidence of PPCs varies considerably across studies, likely due to factors such as the type of surgery and patient–related risk factors, but primarily because there is no uniform definition of this composite outcome.^5 6^

Several attempts have been made to define PPCs, typically expressed as a composite outcome that includes respiratory conditions, need for supplemental oxygen or mechanical ventilation, and complications such as intensive care unit (ICU) readmission and mortality.^5^ The European Perioperative Clinical Outcome (EPCO) definition, developed by a joint ESA–ESICM task force, identified variables for PPCs based on expert opinion but lacked a systematic literature review or formal consensus methodology, raising concerns about group conformity and bias.^7^,^9^ To address these limitations, the Standardised Endpoints for Perioperative Medicine (StEP) collaboration conducted a systematic review of pulmonary outcomes in perioperative patients, synthesising findings to inform a Delphi process.^10^ Unable to reach consensus on a universal definition, StEP proposed grouping PPCs by shared pathophysiology, such as pulmonary collapse and airway contamination.^8^ However, limitations included a lack of representation from low-resource settings, no patient involvement and potential bias due to non-anonymised voting and pre–final round discussions.^11 12^

Composite outcome measures can enhance statistical power by increasing event rates.^7^ However, they can obscure differences among individual components, as definitions often rely on binary outcomes without considering incidence, severity or varying diagnostic criteria. Variables such as respiratory infection, pleural effusion and aspiration pneumonitis differ in clinical significance and pathophysiology, complicating assessment. Inconsistent selection criteria and a lack of a universal definition hinder comparability across studies, potentially distorting reported incidence rates and intervention effects.^5^

When using the composite endpoint PPCs, it is crucial to consider the grading and hierarchical ranking of the individual components.^13^ Grading decisions, often based on experts’ opinions, patient input and economic insights, influence study results, underscoring the need for clear and consistent methodologies. The Clavien-Dindo classification system,^14^ although widely used for grading PPCs, has several limitations. This intervention–focused model has limitations when applied to PPCs, particularly in underestimating less severe complications that may only require observation.^15^ Furthermore, it lacks consistency across surgical specialities and has reduced applicability in low and middle-income countries (LMICs) due to variable resource availability and care practices.^16^ Importantly, it does not account for the long-term impact of complications on patients’ daily life and function.

Not all PPCs are of equal severity or clinical importance; for instance, severe respiratory failure might have more profound implications than a minor pleural effusion. Establishing a hierarchy among PPC components involves evaluating their relative severity and impact on patient outcomes. This ranking can help in interpreting the composite endpoints more meaningfully, distinguishing between minor and major complications.^17^ However, determining this hierarchy is not straightforward and often lacks standardisation.

The main objective of this international Delphi, named ‘Postoperative pulmonary complications—Evaluation for Consensus on Standardised Outcomes’ (PrECiSIOn), is to develop expert consensus on the definition of the composite PPCs and weightage grading of individual PPCs. Secondary objectives are to generate consensus on the timeframe and methods of monitoring PPCs. Specifically, we aim to develop a reliable, valid, feasible and universally accepted patient–centred definition of PPCs.

## Methods

### Design

PrECiSIOn is an international Delphi process that will be convened by a steering committee comprised of healthcare professionals with research experience in PPCs or postoperative respiratory failure, a patient care representative (SB), a health economist (RO) and a Delphi methodologist (PN). A diverse international panel will be invited based on predefined criteria. The members of the steering committee conducted a focused systematic search of the published literature between June 2018 and March 2025, using the PubMed and Embase databases to identify studies that reported PPCs as the primary or secondary outcomes. Our findings indicate that only two out of a total of 29 (7%) studies explicitly adhered to the EPCO or StEP definitions (table 1). The literature search will be used to inform the preliminary list of statements for the Delphi rounds, and the synthesis of the selected articles will be shared with the panellists. The steering committee members will organise iterative Delphi rounds, prepare and review the Delphi report, develop statements for subsequent Delphi rounds and provide controlled feedback to the panellists. The steering committee will not be allowed to vote in the Delphi rounds to avoid any bias.^11 18^ The results of this Delphi study will be reported using ACcurate COnsensus Reporting Document (ACCORD) guidelines.^19^

**Table 1:** Studies identified through a focused systematic search using the postoperative pulmonary complications

Study	Publication year	PMID or DOI	EPCO or StEP	Symptoms	Diagnoses	Therapeutic consequences
He *et al*	2024	39256677			Atelectasis, respiratory failure, suspected or confirmed pneumonia	Prolonged mechanical ventilation, ICU re-admission
Li *et al*	2024	38374814			Atelectasis, pneumonia	Reintubation, need for bronchoscopy, death
Sahin *et al*	2024	10.4274/atfm.galenos.2024.82905		Bronchospasm	Atelectasis, respiratory failure, ARDS, suspected respiratory infection, pneumothorax, aspiration pneumonitis, pleural effusion	
Xuefei *et al*	2024	39278547		Bronchospasm	Atelectasis, respiratory failure, pneumonia, pneumothorax, aspiration pneumonitis, pleural effusion	
Admass *et al*	2023	37255746		Bronchospasm	Atelectasis, respiratory failure, suspected or confirmed pneumonia, ARDS, pneumothorax, aspiration pneumonitis, pulmonary congestion, pleural effusion, tracheobronchitis	
Huang *et al*	2023	37559604			Respiratory failure, suspected or confirmed pneumonia, pleural effusion, pulmonary embolism	
Ramkumar *et al*	2023	10.47009/jamp.2023.5.4.126			Atelectasis, respiratory failure, pneumonia, pleural effusion, pulmonary embolism, exacerbation of COPD	Death
Salling *et al*	2023	36596755			Atelectasis, respiratory failure	
Morris *et al*	2022	36219615			Atelectasis, suspected or confirmed pneumonia, ARDS, pneumothorax, aspiration pneumonitis, pulmonary congestion, pleural effusion, pulmonary embolism, haemothorax, bronchopleural fistula, empyema	Prolonged mechanical ventilation
Chai	2021	33174436				
Ledowski *et al*	2021	34127252			Atelectasis, pneumonia, suspected or confirmed pneumonia	
Wang *et al*	2021	34654446		Bronchospasm	Atelectasis, respiratory failure, ARDS, suspected or confirmed pneumonia, pulmonary congestion, pleural effusion	
Charokar	2020	10.7860/JCDR/2020/45276.13921	EPCO		Atelectasis, respiratory failure, pneumonia, pulmonary congestion, pleural effusion	
Gupta	2020	32174665		Bronchospasm	Atelectasis, pneumonia, aspiration pneumonitis, pleural effusion, pulmonary embolism	
Gülsen *et al*	2020	33101428		Bronchospasm	Atelectasis, respiratory failure, pneumonia, ARDS, pleural effusion	Reintubation, prolonged mechanical ventilation
Karalapillai *et al*	2020	32870298		Bronchospasm	Atelectasis, respiratory failure, pneumonia, pneumothorax, pulmonary congestion, pleural effusion	Prolonged mechanical ventilation
Lee *et al*	2020	32709867		Hemoptysis	Atelectasis, pneumonia, ARDS, pneumothorax, pleural effusion, bronchopleural fistula, chylothorax, empyema	
Pramanik	2020	32346174		Bronchospasm	Respiratory failure, pneumonia, pneumothorax, pulmonary congestion	Prolonged mechanical ventilation, reintubation
Togioka *et al*	2020	32139135			Atelectasis, respiratory failure, pneumonia, pneumothorax, aspiration pneumonitis, upper airway obstruction	Need for invasive or non-invasive ventilation
Zhu *et al*	2020	32631414	EPCO	Bronchospasm	Atelectasis, respiratory failure, ARDS, suspected or confirmed pneumonia, pneumothorax, aspiration pneumonitis, pulmonary congestion, pleural effusion	
Bluth	2019	10.1001/jama.2019.7505		Bronchospasm	Atelectasis, respiratory failure, ARDS, suspected or confirmed pneumonia, pneumothorax, pulmonary congestion, pleural effusion	
Hooda	2019	31303718		Bronchospasm	Atelectasis, respiratory failure, pneumonia, ARDS, pneumothorax, tracheobronchitis	Prolonged mechanical ventilation, reintubation
Vasu	2019	10.4103/ijrc.ijrc_53_18		Bronchospasm	Atelectasis, respiratory failure, pneumothorax, suspected or confirmed pneumonia, pleural effusion	Prolonged mechanical ventilation, reintubation
Kabon	2019	10.1097/ALN.0000000000002601			Respiratory failure, pneumonia, pulmonary congestion, pulmonary embolism	Prolonged mechanical ventilation
Jing	2018	Int J Clin Exp Med 2018;11(1):285–94			Atelectasis, respiratory failure, pneumonia, ARDS, pulmonary congestion	
Kumar	2018	29628582		Bronchospasm	Atelectasis, respiratory failure, pneumonia, pleural effusion	

ARDS, Acute Respiratory Distress Syndrome; COPD, Chronic Obstructive Pulmonary Disease; EPCO, The European Perioperative Clinical Outcome; ICU, intensive care unit; StEP, Standardized Endpoints for Perioperative Medicine.

### Patient and public involvement

Individuals who have either personally experienced PPCs following surgery within the last 5 years or primary caregivers of such patients will be recruited as patient care representatives. Proficiency in English is required, and to mitigate potential bias, it is essential that the patient care representatives do not maintain a professional or advisory relationship with the steering committee or panellists. A concerted effort will be made to ensure a balance of geographical location, socioeconomic background, sex and ethnicity.

After obtaining informed consent, patients or patient care representatives will be engaged through structured interviews conducted by members of the steering committee and an independent qualitative research expert. A pilot-tested case vignette along with a Likert scale-based questionnaire will be employed to gather insights regarding the impact and severity of PPCs. The impact of the individual components of PPCs will be evaluated on a 7-point Global Rate of Change scale (from ‘very much worse’ to ‘very much better’) to calculate the minimal clinically important difference. Patient care representatives will be engaged in anonymous voting on the questionnaire. PPCs ranking and key themes synthesised from the interview will be shared with the panel in the second Delphi round, facilitating integration of patient perspectives into the consensus process. The interview data will be archived for a minimum of 5 years in a cloud server monitored by the Institutional Review Board of the approving organisation.

### Specific objectives

To generate consensus on the PPCs construct;

To generate consensus on the individual components of PPCs and their definition;

To generate consensus on the timeframe of monitoring for PPCs;

To generate consensus on the methods of monitoring PPCs, and

To generate consensus on the weightage grade of individual PPCs.

### Panellists

An international panel of 35–40 panellists from six continents, including patient representatives, will be invited to this Delphi study. A concerted effort will be made to include panellists from LMICs, as well as for an age and sex balance.

Panellists will be identified based on *either of* the following criteria:

1. At least 10 years of clinical experience as a staff member in Anaesthesiology, Surgery or Intensive Care Medicine with involvement in care of patients with postoperative complications.
2. Author of at least three publications (observational studies or randomised clinical trials) using PPCs as a primary or secondary outcome.

The predefined selection criteria will facilitate a balanced composition of panellists possessing clinical or academic expertise in the field of PPCs. This approach will enhance the relevance, reliability and generalisability of the consensus findings. Panellists will be identified through a purposive sampling from the publications on PPCs and meeting the above–mentioned selection criteria. On confirming their acceptance, the panellists will be provided with a succinct summary of the Delphi process, explaining the role and objectives, expected schedule of Delphi surveys and duration required to complete each survey. To encourage continuing engagement of the panellists and to help prevent them from dropping out before completion of the full process, at least three personalised email reminders will be sent during each Delphi round, in addition to an effort to establish personal contact. Furthermore, all panellists will be recognised as collaborative authors in the final publication in appreciation of their professional expertise. Should the attrition rate of panellists exceed 20%, a sensitivity analysis will be conducted with the plausible worst–case and best–case scenarios, considering the opinion of non-responders.

### Delphi rounds

**Figure 1:**
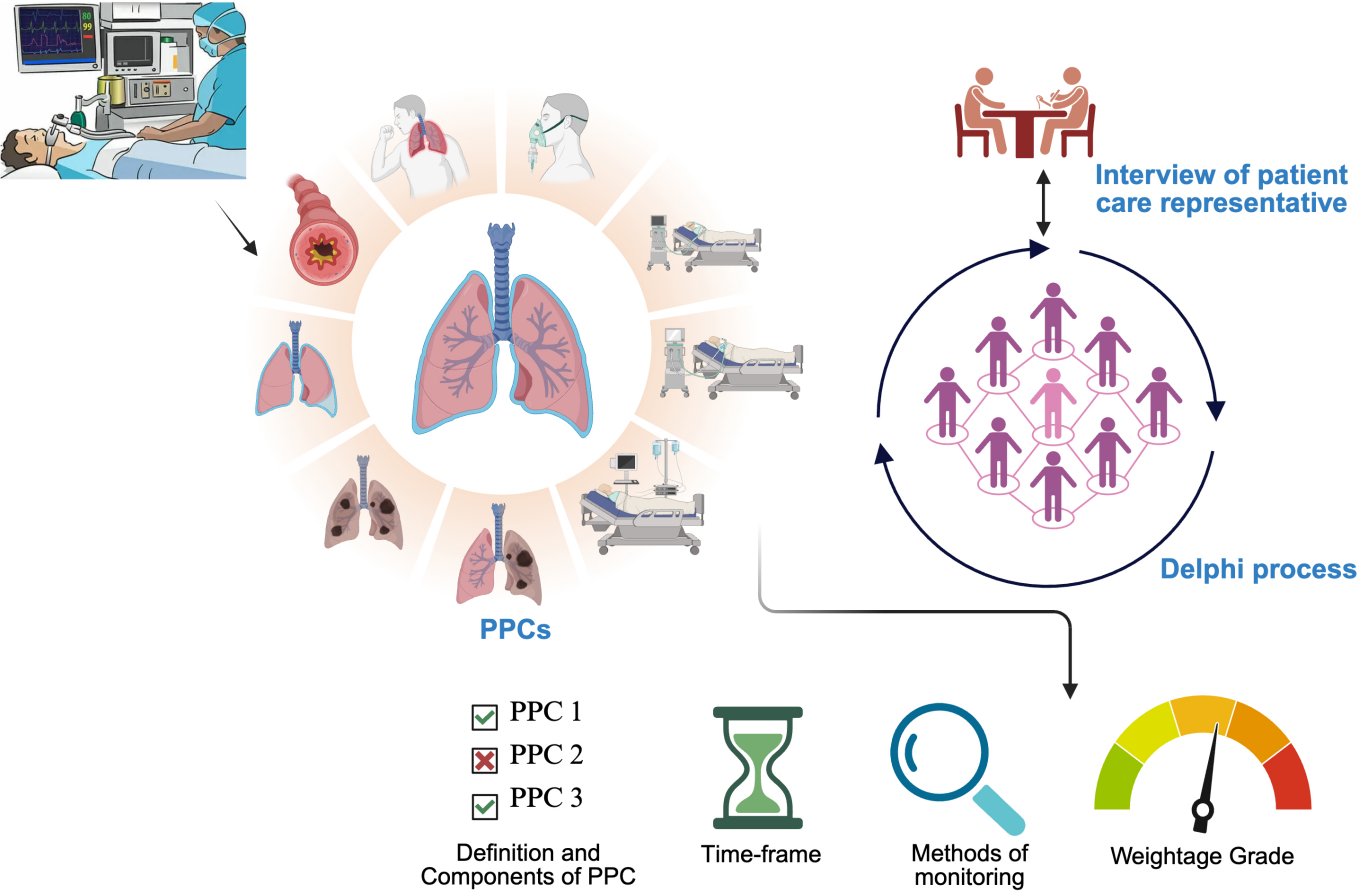
Steps of the Delphi process employed for the study. PPCs, Postoperative pulmonary complications, created in BioRender.com.

Panellists and their responses will remain anonymised during the Delphi Rounds. Search results will inform the opening statements of the Delphi Rounds. The statements will be prepared on Google Forms as multiple–choice or 7-point Likert scales. Delphi round surveys will be presented to panellists through a unique online link. Multiple iterative Delphi rounds will be conducted until stable responses are achieved for all statements. Delphi round reports and patient–centred outcomes obtained from the interviews and questionnaire, as well as anonymised cumulative feedback, will be shared with panellists (figure 1).

### Consensus and stability

Consensus for this Delphi study will be defined as ≥75% agreement (or disagreement) on the Likert scale, and for option(s) in multiple-choice statements. Stability of the responses will be calculated through non-parametric χ^2^ tests for multiple–choice statements and Kruskal-Wallis tests for Likert scale statements from round 2 onwards. A p value <0.05 will represent significant variation in the responses, and the statement will be repeated in the subsequent Delphi round. Open-ended responses from participants will be analysed using thematic analysis to identify recurring ideas and insights. Two independent researchers will code the data, with discrepancies resolved by discussion or a third reviewer. Open–text comments to survey questions will inform revisions to the questions for the subsequent Delphi rounds. Where more than 10% of responses include congruent comments on a particular item, the item will be modified for subsequent Delphi rounds.

### Consensus process

This study adopts a mixed-methods consensus design to ensure comprehensive stakeholder involvement. Two parallel methodologies will be employed:

Nominal group technique (NGT): to gather and prioritise perspectives from patients and patient care representatives, a structured NGT approach will be used. This technique facilitates in–depth discussion and the ranking of PPCsin a virtually moderated setting. It ensures that diverse lived experiences are reflected and incorporated into the prioritisation process.

Modified Delphi methodology: for consensus among panellists, a modified Delphi process will be undertaken. This will involve multiple anonymous iterative survey rounds, enabling participants to rate and revise their responses based on group feedback.

### Final consensus

The stable statements from Delphi rounds will inform the consensus definition, components, timeframe and appropriate monitoring of PPCs and will be vetted by panellists before publishing.

### Weightage grade

PPCs will be further categorised, ranging from 0–6, with 0 (normal health without disability) to 6 (dead or as bad as being dead).^20^ Panellists will be asked to provide weightage grade for categories 1 to 5 using the WHO Global Burden of Disease Project (WHOGBDP) person trade-off method in the special Delphi rounds for weightage grade.^21^ As a reference framework for this task, the panel members will be provided with the WHOGBDP framework table, clinical vignettes displaying seven disability classes and 22 anchoring example conditions. During the initial Delphi rounds, panellists will also be asked to establish consensus on the relevance and use of the WHOGBDP–based severity grading system for PPCs. The coefficient of variation (CV) will be used to determine the need for additional Delphi rounds, with CV ≤0.5 indicating achievement of substantial consensus.

## Discussion

Most studies that use PPCs as endpoints have employed composite measures that combine respiratory conditions with the need for supplemental oxygen or mechanical ventilation. Some studies have also included non–pulmonary PPCs, like sepsis, unplanned admission to an ICU, hospital readmission and mortality.^5^ The EPCO definition identified variables related to the composite outcome of PPCs based on the limited available literature suggested by the opinion leaders.^7^ However, the panel did not conduct a systematic search for existing definitions and did not use a formal, structured consensus method, which raised concerns regarding bias due to limited perspectives and group conformity.^9^

Subsequently, the StEP collaboration under the aegis of the British Journal of Anaesthesia was commissioned to create consensus–based definitions and outcome measures for perioperative medicine.^10^ They conducted a systematic literature search of studies on pulmonary outcomes in perioperative patients, and the findings from selected articles were synthesised to create a preliminary list of outcome variables and their definitions. The list was then used to inform a Delphi process to generate consensus on the variables of composite outcomes. However, they were unable to reach a consensus on a universal definition and proposed a new definition for the composite diagnosis of PPCs encompassing conditions with shared pathophysiology.^8^ Notwithstanding the involvement of an expert panel, considerable limitations were apparent in their Delphi process. There was a conspicuous absence of panellists from LMICs, as well as resource–limited settings, and a lack of involvement of the patients or patient care representatives. Furthermore, panellists were not anonymised and an open discussion among the panellists occurred before the final round of voting, which may have confounded the results due to influence of peer pressure and a potential dominance bias.^11 18^ Finally, our literature review revealed a limited adoption of established PPC definitions and notable variability in the composite outcomes used, as well as in the definitions of the individual components of PPC.

The current Delphi study aims to critically evaluate and refine the proposed framework of PPCs by addressing several key methodological and practical considerations. The fundamental question of whether the proposed framework of a composite outcome for PPCs constitutes a valid hypothetical construct is essential for ensuring its applicability in clinical and research settings. Construct validity depends on whether the identified PPCs accurately reflect postoperative pulmonary morbidity and whether they align with existing clinical knowledge and outcomes. Our study will use a robust and transparent Delphi process to assess the opinion of a diverse international panel of experts on the construct of PPCs and to further assess whether the components included in the framework have sufficient empirical support and consensus within the medical community and researchers in the field. The engagement of an international diverse panel, including patient or patient care representatives as well as health economists, will facilitate the refinement of the definition and selection of patient–centred PPCs. This approach aims to enhance the generalisability and applicability of the definition in both research and clinical practice.

Achieving agreement on the individual components is critical to standardisation and comparability across studies. Each PPC included should be independently meaningful, clinically significant and contribute uniquely to patient outcomes. The methods of monitoring and interventions, such as high–flow nasal oxygen or non–invasive ventilation, may alter the presentation of some PPCs, potentially leading to underreporting or misclassification.^5^ This study will analyse the appropriate definition and whether interventions complicate the identification and classification of PPCs and propose strategies to mitigate these challenges.

The composite outcome of PPCs may provide a generally acceptable and practical representation of postoperative pulmonary morbidity by encompassing multiple adverse events. However, it also presents challenges, including the risk of masking individual event significance and diluting clinically meaningful differences. There have been attempts to categorise severity of PPCs.^8 14^ However, challenges arise in defining clear and universally applicable criteria. For instance, mild atelectasis seen on imaging may not always be clinically significant, whereas respiratory failure requiring reintubation clearly represents a severe complication. Furthermore, prioritisation of PPCs may enhance focus on the most impactful complications. This study will determine whether ranking PPCs based on severity, prevalence or patient outcomes is beneficial and whether a weighted scoring system might be warranted. The study will assess whether a standardised severity classification is feasible and beneficial. This Delphi study will adopt the WHOGBDP disability weighting framework to classify the severity of PPCs.^21^ The GBD scale (0–6) is designed to reflect the degree of functional health loss and disability associated with specific health states, providing a consistent, patient–centred and outcome–based approach. Furthermore, we aim to generate consensus on the weightage grade of individual components of PPCs.

The chosen timeframe for assessing PPCs significantly influences the reported incidence and severity of complications. There is a lack of consensus on the appropriate timeframe for detecting PPCs, which is a clear challenge to research in this field. A 5–day window aligns with early postoperative pulmonary morbidity, yet some complications, such as pneumonia, may manifest later.^22^ To address this, a flexible approach will be adopted during the consensus process: panellists will be asked to reflect on the appropriateness of the 5–day window and to provide input on whether extended monitoring periods may be necessary for specific high–risk subgroups. These considerations will help determine whether future definitions should recommend context–specific follow-up durations.

### Ethics and dissemination

 The study will be conducted in full concordance with the principles of the Declaration of Helsinki and will be reported according to ACCORD guidance. This study has been granted ethical approval by the University of Wolverhampton (SOABE/202425/staff/3). The key ethical considerations of the study are peer pressure and group conformity. To mitigate this, the experts will remain anonymous to each other until the end of the Delphi process. Informed consent will be obtained from all panellists before the start of the Delphi process. The study will be published in a peer–reviewed journal with the authorship agreed as per ICMJE requirements. The study protocol is also registered and available at clinicaltrials.gov (identifier NCT NCT06916598).
