# Supplementary material for: Consensus on the definition, components, timeframe and grading of composite outcome of postoperative pulmonary complication—protocol for an international mixed-method consensus study (PrECiSIOn)
Source: BMJ Open. 2025 Aug 19;15(8):e103888. doi: 10.1136/bmjopen-2025-103888 (PMC12366572; doi:10.1136/bmjopen-2025-103888)
Supplement: Supplementary file 1 [file bmjopen-2025-103888.reviewer_comments.pdf]

PEER REVIEW HISTORY

BMJ Open publishes all reviews undertaken for accepted manuscripts. Reviewers are asked to complete a checklist review form and are provided with free text boxes to elaborate on their assessment. These free text comments are reproduced below.

ARTICLE DETAILS

Title (Provisional)

Consensus on the Definition, Components, Timeframe and Grading of Composite Outcome of Postoperative Pulmonary Complication— protocol for an International Mixed-method Consensus study (PrECiSIOn)

Authors

Nasa, Prashant; Yurttas, Timur; Battaglini, Denise; Blot, Stijn; Fernandez-Bustamante, Ana; Gama de Abreu, Marcelo; van Meenen, David M; Myatra, Sheila Nainan; Serpa Neto, Ary; Oppong, Ray; Paulus, Frederique; Renukappa, Suresh; Schultz, Marcus J; Slutsky, Arthur S.; Hemmes, Sabrine N. T.; for PrECiSIOn, group

---

VERSION 1 - REVIEW

Reviewer 1

Name Lei, Chong

Affiliation Fourth Military Medical University, Department of Anesthesiology and Perioprative Medicine, Xijing Hospital

Date 18-May-2025

COI None

This protocol outlines a well-structured Delphi study aimed at establishing consensus on the definition, components, and severity grading of postoperative pulmonary complications (PPCs). This protocol aimed to address the inconsistent definitions of PPCs, which now hindered comparability across studies and limit evidence-based perioperative care.

1.Inclusion of clinicians, researchers, patients, and health economists enhances the validity and generalizability of the consensus. Efforts to recruit panellists from low- and middle-income countries (LMICs) are commendable. However, the panellists are required to have ≥10 years of clinical experience or three PPC-related publications, the rationale for these thresholds is unclear. A justification based on expertise relevance (e.g., PPC management vs. research) would strengthen transparency.

2.There are two definition of PPCs estabolished, that is EPCO definition and StEP definition. the authors listed some of the limitations of these definitions. If detailed data can be provided to demonstrated the use of these two definitions in current studies was low

(indicated that these two definitions were not well adopted), that will help to validated the need for conducting this study and to estabolished a new definition for PPCs.

3.Structured interviews with patients and caregivers ensure that the final definition reflects patient priorities, a critical yet often overlooked aspect of outcome standardization. However, While No details are provided about how patient representatives will be selected or how their input will be weighted against clinician/researcher opinions. Please define criteria for patient representative inclusion (e.g., prior PPC experience, demographic diversity) and explain how their input will be integrated with expert opinions.

4.In addition, I suggest to include the minimal clinical important difference (MCID) for PPCs for faciliate future study design.

5.The protocol mentions "periodic reminders" to retain panellists but lacks specifics (e.g., frequency, follow-up strategies for non-responders). Specify contingency plans (e.g., replacement panellists, statistical adjustments) if attrition exceeds 20%.

6.The WHO Global Burden of Disease (WHOGBDP) framework for grading PPC severity (0–6 scale) is appropriate but requires clearer operationalization. For example, how will panellists reconcile subjective interpretations of "disability" across different healthcare settings? Currently, the severity of PPCs are value based on Clavien-Dindo score, please specify the difference of these two score system.

7.The rationale for selecting a 5-day postoperative monitoring window (vs. longer periods) is not discussed, despite evidence that complications like pneumonia may manifest later. Discuss trade-offs between shorter vs. longer monitoring periods and propose a flexible approach (e.g., 5-day minimum with extended follow-up for high-risk patients).

Reviewer **2**

Name **Nurmatov, Ulugbek**

Affiliation **Division of Population Medicine, School of Medicine, Cardiff University**

Date **07-Jun-2025**

COI **None**

This study aims to develop a universally accepted framework for understanding postoperative pulmonary complications.

The paper is well-structured and written, and it is easy to read. There are, however, a number of issues to address:

1. The study method is complex. What type of study is it (clinical trial, observational cohort study, qualitative study, a combination of Nominal Group Technique (NGG) and Delphi or a

modified e-Delphi study)? If it is a mixed/modified e-Delphi study, can you please clarify and provide relevant references to support it?

2. While you have the term “Respiratory Distress Syndrome” in Keywords, you never mentioned it in the body of the protocol.

3. How do you handle the qualitative analysis: open-ended questions, individual feedback, qualitative synthesis method, sentiment analysis, scenario analysis etc.?

4. How are you going to recruit international 40 experts (source of panelists)? Interactions between Expert Panel members (professionals) via Questionnaires and interviews with patients and representatives?

5. What are the termination criteria?

6. Please provide the advantages of the 7-point scale vs 4-point scale. A 4-point Likert scale has demonstrated efficacy and produced stable findings (Vogel 2019; Akins 2005). In addition, it will remove ambiguity of responses e.g. “Neither agree nor disagree, the respondent has no opinion”

Overall, this is an important study that will have the potential to impact the lives of patients with PPCs, practice, and policy.

VERSION 1 - AUTHOR RESPONSE

Reviewer: 1

This protocol outlines a well-structured Delphi study aimed at establishing consensus on the definition, components, and severity grading of postoperative pulmonary complications (PPCs). This protocol aimed to address the inconsistent definitions of PPCs, which now hindered comparability across studies and limit evidence-based perioperative care.

1. Inclusion of clinicians, researchers, patients, and health economists enhances the validity and generalizability of the consensus. Efforts to recruit panellists from low- and middle-income countries (LMICs) are commendable. However, the panellists are required to have ≥10 years of clinical experience or three PPC-related publications, the rationale for these thresholds is unclear. A justification based on expertise relevance (e.g., PPC management vs. research) would strengthen transparency.

Thank you for your insightful comment. To ensure the methodological rigor and credibility of the Delphi process, we selected two thresholds for expert panel inclusion: ≥10 years of relevant clinical experience and/or ≥3 PPC–related publications. The ≥10 years cutoff serves as a proxy for sustained senior–level involvement in perioperative care and management of PPCs. Clinical experience ensures that panellists bring deep, practice–based insights grounded in real–world patient care. The publication criterion was selected to reflect ongoing and focused academic contribution to the field, rather than incidental involvement.

These cutoffs were informed by standards used in prior Delphi studies and aimed at ensuring sufficient subject–matter expertise without being overly exclusive (doi: 10.1186/s12909-025-07520-0, doi: 10.1186/1745-6215-13-176).

We explain this in the revised version of the manuscript, as follows:
The predefined selection criteria will facilitate a balanced composition of panellists possessing clinical or academic expertise in the field of PPCs. This approach will enhance the relevance, reliability, and generalisability of the consensus findings. **(Page 12; line 9)**

2. There are two definitions of PPCs established, that is EPCO definition and StEP definition. the authors listed some of the limitations of these definitions. If detailed data can be provided to demonstrated the use of these two definitions in current studies was low (indicated that these two definitions were not well adopted), that will help to validated the need for conducting this study and to established a new definition for PPCs.

Thank you for your thoughtful comment and for highlighting the importance of demonstrating the limited adoption of existing PPC definitions.

Indeed, we conducted a focused literature review of studies published between June, 2018 to March, 2025 that reported postoperative pulmonary complications (PPCs) as either a primary or secondary outcome. Our findings indicate that only two out of total 29 (7%) studies explicitly adopted the EPCO or StEP definitions. The majority of the studies employed a variety of outcomes, demonstrating significant discrepancies in the descriptions of these components or undefined descriptors of PPCs, reflecting a lack of standardisation across the literature.

We have presented this data in Table 1, summarising the definitions used, publication year, study design. This reinforces the argument that existing definitions have not seen widespread clinical or research uptake.

We believe this evidence further validates the need for our current study, which seeks to establish a consensus–driven, clinically applicable, and widely acceptable definition of PPCs.

We have made the following changes in the revised version of the manuscript accordingly:

Methods
The members of the steering committee conducted a focused systematic search of the published literature between June, 2018 to March, 2025 utilising the PubMed and Embase databases to identify studies that reported PPCs as the primary or secondary outcomes. Our findings indicate that only two out of a total of 29 (7%) studies explicitly adhered to the EPCO or StEP definitions (Table 1). **(Page 10; line 5)**

Discussion

Finally, our literature review revealed a limited adoption of established PPCs definitions, and notable variability in the composite outcomes used, as well as in the definitions of the individual components of PPC. **(Page 16; line 25)**

3. Structured interviews with patients and caregivers ensure that the final definition reflects patient priorities, a critical yet often overlooked aspect of outcome standardization. However, While No details are provided about how patient representatives will be selected or how their input will be weighted against clinician/researcher opinions. Please define criteria for patient representative inclusion (e.g., prior PPC experience, demographic diversity) and explain how their input will be integrated with expert opinions.

Thank you for this comment and suggestion. To ensure that patient perspectives meaningfully contribute to the development of consensus on PPCs, the following inclusion criteria will be used to recruit patient representatives:

1. **Personal Experience with PPCs: Individuals who have experienced PPCs themselves following surgery (e.g., pneumonia, atelectasis, respiratory failure) within the last 5 years. Alternatively, primary caregivers of such patients may also be considered to capture the carer's perspective.**
2. **Ability to Participate in English-Language Discussion: As the Delphi rounds and communication will be conducted in English, basic proficiency in English is necessary.**
3. **No Prior Professional Affiliation with the Study Team: To reduce potential bias, patients or caregivers with a professional or advisory relationship to the steering committee or panellist will be excluded.**
4. **Diverse Demographic Representation: Efforts will be made to include patients from a range of geographic regions, socioeconomic backgrounds, age groups, sex, and ethnicities to reflect the global patient population and ensure varied perspectives.**

We have included further details about the recruitment of patient care representative as follows:
Individuals who have either personally experienced PPCs following surgery within the last 5 years or, primary caregivers of such patients will be recruited as patient care representatives. Proficiency in English is required, and to mitigate potential bias, it is essential that the patient care representatives do not maintain a professional or advisory relationship with the steering committee or panellists. A concerted effort will be made to ensure a balance of geographical location, socioeconomic background, sex, and ethnicity. **(Page 10; line 17)**

4.In addition, I suggest to include the minimal clinical important difference (MCID) for PPCs for facilitate future study design.

Thank you for this suggestion. We will evaluate the variables in composite outcome for minimal clinical important difference through a 7-point global rate of change scale (from "very much worse" to very much better").
We explain this in the revised version of the manuscript, as follows:

A pilot–tested case vignette along with a Likert scale–based questionnaire will be employed to gather insights regarding the impact and severity of PPCs. The impact of the individual components of PPCs will be evaluated on a 7-point Global Rate of Change (GRC) scale (from "very much worse" to very much better") to calculate the minimal clinical important difference. Patient care representatives will be engaged in anonymous voting on the questionnaire. **(Page 11; line 5)**

5.1 The protocol mentions "periodic reminders" to retain panellists but lacks specifics (e.g., frequency, follow-up strategies for non-responders).

Thank you for the comment. A range of proactive strategic measures will be used to retain panellists. After confirming their acceptance to the invitation email, the panellists will be provided with a comprehensive and succinct overview of the methodology. This will outline their role and objectives, the anticipated schedule for the surveys, and the expected duration for completion of each Delphi round. A minimum of three personalised email reminders will be sent during each Delphi round, in addition to an effort to establish personal contact, reinforcing the study timelines and the significance of their contribution. Furthermore, all panellists will be recognised as collaborative authors in the manuscript in appreciation of their professional expertise.
We have clarified this in the methods as follows:
Upon confirming their acceptance, the panellists will be provided a succinct summary of the Delphi process, explaining the role and objectives, expected schedule of Delphi surveys and duration required to complete each survey. To encourage continuing engagement of the panellists and to help prevent them from dropping out before completion of the full process, at least three personalised email reminders will be sent during each Delphi round, in addition to an effort to establish personal contact. Furthermore, all panellists will be recognised as collaborative authors in the final publication in appreciation of their professional expertise. **(Page 12; line 13)**

5.2 Specify contingency plans (e.g., replacement panellists, statistical adjustments) if attrition exceeds 20%.

Thank you for your comment. We will invite 10% more panellists than our target for this Delphi study. In the event that the attrition rate of panellists exceeds 20%, a sensitivity analysis will be conducted, comparing the results to plausible worst–case and best–case scenarios considering the opinion of non–responders.
We have included this in the revised version of the manuscript as follows.
Should the attrition rate of panellist exceeds 20%, a sensitivity analysis will be conducted with the plausible worse–case and best–case scenario, considering the opinion of non-responders. **(Page 12; line 21)**

6.The WHO Global Burden of Disease (WHOGBDP) framework for grading PPC severity (0–6 scale) is appropriate but requires clearer operationalization. For example, how will panellists reconcile subjective interpretations of "disability" across different healthcare settings? Currently, the severity of PPCs are value based on Clavien-Dindo score, please specify the difference of these two score system.

Thank you for this thoughtful comment. Although, Clavien-Dindo classification, is widely used for grading PPCs, it presents several limitations when applied to PPCs. Specifically, it may underestimate less severe complications that may require close monitoring, but no active therapeutic interventions (doi: 10.1097/DCR.0000000000001966). Furthermore, its applicability can vary across surgical specialities and diverse healthcare settings, particularly in low–middle income countries due to resource constraints and differing care practices (doi: 10.1002/bjs.11025). Importantly, it does not capture the impact of complications on patient's daily functioning or long–term health outcomes.

The WHO Global Burden of Disease (WHOGBD) framework was selected as it provides a consistent and internationally recognised method for grading health states based on their associated disability. Unlike the Clavien-Dindo classification, which grades complications based on the therapeutic interventions (e.g., pharmacological, surgical, ICU admission), the WHO GBD framework assigns scores based on the degree of health loss and functional impairment (0–6 scale), thereby offering a patient–centred and outcome–based perspective.

To mitigate variation in interpretation of "disability" across diverse healthcare contexts, we will provide panellists with clear operational definitions, clinical examples, and anchoring scenarios to support consistent scoring. In addition, we will seek consensus on the relevance and application of severity levels for PPCs during the initial Delphi rounds to ensure a shared understanding among the panel.

We have incorporated this rationale in the revised version of the manuscript as follows:
Introduction
The Clavien-Dindo classification system [14], although widely used for grading PPCs, has several limitations. This intervention–focused model has limitations when applied to PPCs, particularly in underestimating less severe complications that may only require observation [15]. Furthermore, it lacks consistency across surgical specialties and has reduced applicability in low– and middle–income countries due to variable resource availability and care practices [16]. Importantly, it does not account for the long–term impact of complications on patients' daily life and function. **(Page 8; line 10)**

Methods
As a reference framework for this task, the panel members will be provided with the WHOGBDP framework table, clinical vignettes displaying seven disability classes and 22 anchoring example conditions. During the initial Delphi rounds, panellists will also be asked to establish consensus on the relevance and use of the WHOGBD–based severity grading system for PPCs. **(Page 14; line 17)**

Discussion
This Delphi study will adopt the WHOGBD disability weighting framework [21] to classify the severity of PPCs. The GBD scale (0–6) is designed to reflect the degree of functional health loss and disability associated with specific health states, providing a consistent, patient–centred, and outcome–based approach. **(Page 18; line 15)**

7.The rationale for selecting a 5-day postoperative monitoring window (vs. longer periods) is not discussed, despite evidence that complications like pneumonia may manifest later. Discuss trade-offs between shorter vs. longer monitoring periods and propose a flexible approach (e.g., 5-day minimum with extended follow-up for high-risk patients).

Thank you for this important comment. Indeed, we agree that the timing of PPCs can vary, and complications such as pneumonia and ARDS may occur beyond the immediate postoperative period. The decision to focus on a 5–day postoperative window was informed by previous studies demonstrating that the majority of PPCs develop within the first 48–120 hours post-surgery, particularly in patients undergoing major abdominal or thoracic procedures (doi: 10.1016/j.jss.2015.03.028, doi: 10.1097/ALN.0b013e3181fc6e0a).
We recognise that this fixed time frame may not capture all late–onset complications and could limit applicability across diverse patient populations. To address this, a flexible approach has been adopted: while the 5–day window will serve as the primary reference for defining and assessing PPCs, panellists will be invited to comment on the appropriateness of this timeframe and to consider extended monitoring for high–risk subgroups. This feedback will be incorporated into the consensus process and may inform recommendations for context–specific follow–up durations in future PPC definitions.

We have included this in the Discussion of the revised manuscript as follows:
A 5–day window aligns with early postoperative pulmonary morbidity, yet some complications, such as pneumonia, may manifest later [24]. To address this, a flexible approach will be adopted during the consensus process: panellists will be asked to reflect on the appropriateness of the 5–day window and to provide input on whether extended monitoring periods may be necessary for specific high–risk subgroups. These considerations will help determine whether future definitions should recommend context–specific follow–up durations. **Page 18; line 23)**

Reviewer: 2
Comments to the Author:

This study aims to develop a universally accepted framework for understanding postoperative pulmonary complications. The paper is well-structured and written, and it is easy to read.

Thank you!

There are, however, a number of issues to address:
1. The study method is complex. What type of study is it (clinical trial, observational cohort study, qualitative study, a combination of Nominal Group Technique (NGG) and Delphi or a modified e-Delphi study)? If it is a mixed/modified e-Delphi study, can you please clarify and provide relevant references to support it?

Thank you for this valuable comment. This study follows a mixed–methods consensus approach. To ensure inclusive and representative input, consensus from patient and patient care representatives will be obtained using the Nominal Group Technique (NGT), which allows structured in–person or virtual discussion to prioritise perspectives. In parallel, consensus among panellist using a modified Delphi methodology, conducted over multiple iterative rounds to refine and converge expert opinion anonymously.

This combined approach allows integration of both experiential knowledge from patient stakeholders and evidence–informed judgments from subject matter experts.

We have clarified this in the Methods of the revised version of the manuscript as follows:

Consensus Process

This study adopts a mixed-methods consensus design to ensure comprehensive stakeholder involvement. Two parallel methodologies will be employed:

Nominal Group Technique (NGT): To gather and prioritise perspectives from patients and patient care representatives, a structured NGT approach will be used. This technique facilitates in–depth discussion and the ranking of PPCs virtually in a moderated setting. It ensures that diverse lived experiences are reflected and incorporated into the prioritisation process.

Modified Delphi Methodology: For consensus among panellists, a modified Delphi process will be undertaken. This will involve multiple anonymous iterative survey rounds, enabling participants to rate and revise their responses based on group feedback. **(Page 13, line 22)**

2. While you have the term “Respiratory Distress Syndrome” in Keywords, you never mentioned it in the body of the protocol.

Apology for this oversight. We have removed “respiratory distress syndrome” as a keyword.

3. How do you handle the qualitative analysis: open-ended questions, individual feedback, qualitative synthesis method, sentiment analysis, scenario analysis etc.?

Thank you for this insightful comment. We plan to handle the qualitative data from open–ended questions and individual feedback using a rigorous thematic analysis approach. This will involve systematic coding of textual responses, followed by the identification and categorisation of recurrent patterns and themes.

To ensure trustworthiness and credibility, two independent researchers will code the responses, with discrepancies resolved through discussion or by a third reviewer if needed.

Open–text responses will be synthesised and summarised to inform revisions to survey questions for subsequent Delphi rounds. A modification threshold will be applied: if >10% of participants provide congruent comments on a specific item, that item will be considered for revision. We will also consider using

elements of qualitative synthesis methods to integrate the insights from diverse stakeholders, including individual reflections and feedback.

This has been included in the methods of the revised manuscript as follows: *Open-ended responses from participants will be analysed using thematic analysis to identify recurring ideas and insights. Two independent researchers will code the data, with discrepancies resolved by discussion or a third reviewer. Open–text comments to survey questions will inform revisions to the questions for the subsequent Delphi rounds. Where more than 10% of responses include congruent comments on a particular item, the item will be modified for subsequent Delphi rounds.* **(Page 13, line 15)**

4a How are you going to recruit international 40 experts (source of panelists)?

Thank you for this question. Panellists will be identified through purposive sampling based on their authorship of publications related to PPCs and fulfilment of predefined selection criteria. Approximately 45 potential panellists (including an additional 10% to account for non–responders) will be invited via email to ensure a final panel of at least 40 international experts.

4b Interactions between Expert Panel members (professionals) via Questionnaires and interviews with patients and representatives?

Thank you for your comment. Structured qualitative interviews with patients and patient representatives will be conducted between the first and second Delphi rounds. This will facilitate in–depth discussion and the ranking of PPCs virtually in a moderated setting. PPCs ranking and key themes synthesised from the interview will be shared with the panel as a summary to inform their responses in the second Delphi round, facilitating integration of patient perspectives into the consensus process.
We have included this in the methods of the revised manuscript as follows: *After obtaining informed consent, patients or patient care representatives will be engaged through structured interviews conducted by members of the steering committee and an independent qualitative research expert. A pilot–tested case vignette along with a Likert scale–based questionnaire will be employed to gather insights regarding the impact and severity of PPCs. The impact of the individual components of PPCs will be evaluated on a 7-point Global Rate of Change (GRC) scale (from “very much worse” to very much better”) to calculate the minimal clinical important difference. Patient care representatives will be engaged in anonymous voting on the questionnaire. PPCs ranking and key themes synthesised from the interview will be shared with the panel in the second Delphi round, facilitating integration of patient perspectives into the consensus process.* **(Page 11, line 3)**

5. What are the termination criteria?

Thank you for this comment. The termination criteria for the Delphi process will be stability of responses for all statements. We have explained this in the Methods section as:

Multiple iterative Delphi rounds will be conducted until stabile responses are achieved for all statements. **(Page 13, line 5)**

6. Please provide the advantages of the 7-point scale vs 4-point scale. A 4-point Likert scale has demonstrated efficacy and produced stable findings (Vogel 2019; Akins 2005). In addition, it will remove ambiguity of responses e.g. “Neither agree nor disagree, the respondent has no opinion”

Thank you for this insightful comment. We have opted to use 7–point Likert scale in this Delphi study because higher order scale (more than 5 point) are particularly valuable in Delphi process, where subtle differences in judgement can significantly contribute to achieving a nuanced consensus. (DOI: 10.1177/20597991231179393) Moreover, the 7–point scale incorporates a central "neutral" option allowing participants to convey ambivalence without feeling compelled to align with either agreement or disagreement. This feature enhances the authenticity of responses and improves the overall quality of the data collected.

Overall, this is an important study that will have the potential to impact the lives of patients with PPCs, practice, and policy.

Thank you for your constructive feedback.

VERSION 2 - REVIEW

**Reviewer**	**1**
**Name**	**Lei, Chong**
**Affiliation**	**Fourth Military Medical University, Department of Anesthesiology and Perioprative Medicine, Xijing Hospital**
**Date**	**14-Jul-2025**
**COI**	

I am happy to read the revised protocol. No more question.
